# Intrauterine protein restriction combined with early postnatal overfeeding was not associated with adult-onset obesity but produced glucose intolerance by pancreatic dysfunction

**DOI:** 10.1186/1743-7075-10-5

**Published:** 2013-01-10

**Authors:** Grazielle Vitória Ponti Coutinho, Felipe Rodrigues Coutinho, Jaline Zandonato Faiad, Marina Satie Taki, Silvia Regina de Lima Reis, Letícia Martins Ignácio-Souza, Adriene Alexandra Paiva, Márcia Queiroz Latorraca, Maria Helena Gaíva Gomes-da-Silva, Maria Salete Ferreira Martins

**Affiliations:** 1 Mestrado em Biociências, Faculdade de Nutrição, Universidade Federal de Mato Grosso, Cuiabá, Mato Grosso, Brazil; 2 Laboratório de Avaliação Biológica de Alimentos, Faculdade de Nutrição, Universidade Federal de Mato Grosso, Cuiabá, Mato Grosso, Brazil; 3 Departamento de Alimentos e Nutrição, Faculdade de Nutrição, Universidade Federal de Mato Grosso, Cuiabá, Mato Grosso, Brazil

**Keywords:** Intrauterine protein restriction, Postnatal overfeeding, Obesity, Visceral fat, Glucose tolerance

## Abstract

We investigated if whether intrauterine protein restriction in combination with overfeeding during lactation would cause adult-onset obesity and metabolic disorders. After birth, litters from dams fed with control (17% protein) and low protein (6% protein) diets were adjusted to a size of four (CO and LO groups, respectively) or eight (CC and LC groups, respectively) pups. All of the offspring were fed a diet containing 12% protein from the time of weaning until they were 90 d old. Compared to the CC and LC groups, the CO and LO groups had higher relative and absolute food intakes, oxygen consumption and carbon dioxide production; lower brown adipose tissue weight and lipid content and greater weight gain and absolute and relative white adipose tissue weight and absolute lipid content. Compared with the CO and CC rats, the LC and LO rats exhibited higher relative food intake, brown adipose tissue weight and lipid content, reduced oxygen consumption, carbon dioxide production and spontaneous activity, increased relative retroperitoneal adipose tissue weight and unaltered absolute white adipose tissue weight and lipid content. The fasting serum glucose was similar among the groups. The area under the glucose curve was higher in the LO and CO rats than in the LC and CC rats. The basal insulinemia and homeostasis model assessment of insulin resistance (HOMA-IR) were lower in the LO group than in the other groups. The total area under the insulin curve for the LO rats was similar to the CC rats, and both were lower than the CO and LC rats. K_itt_ was higher in the LO, LC and CO groups than in the CC group. Thus, intrauterine protein restriction followed by overfeeding during lactation did not induce obesity, but produced glucose intolerance by impairing pancreatic function in adulthood.

## Introduction

Obesity is one of the most daunting health challenges of this century. The obesity epidemic is a major health concern because obesity significantly increases the risk for a variety of chronic metabolic diseases, such as type 2 diabetes, hypertension, hypercholesterolemia and heart disease [1,2]. Epidemiologic studies show a higher incidence of obesity among men whose mothers experienced food deprivation in pregnancy during the Dutch famine in the Second World War [3]. Data have shown that nutritional and hormonal status in early life permanently changes the structure and function of body tissues and systems. In addition, overfeeding after birth may increase the risk of obesity and metabolic disorders. Early postnatal overnutrition can be induced by litter size reduction, which is an appropriate experimental model to study short- and long-term consequences of childhood obesity. Animals raised in a small litter have been shown to develop hyperphagia, hyperinsulinemia, hyperleptinemia and hypertension [4-6], as well as higher total and visceral fat mass, lower HDL-C and central leptin resistance in adult life [7].

Adipose tissue has been shown to play a crucial role in metabolic disorders associated with obesity [8]. Research has demonstrated associations among the location of fat storage, insulin sensitivity and the concurrent impacts on multiple other metabolic complications. Insulin resistance and metabolic syndrome are established consequences of expanding visceral fat mass [9], whereas the expansion of subcutaneous fat is associated with improved glucose tolerance and a lower risk of developing obesity related co-morbidities [10,11]. Moreover, recent studies have shown that higher gluteofemoral fat mass is associated with protection against insulin resistance in obese men and women [12,13]. The protective role of gluteofemoral body fat results from its distinct properties with regard to lipolysis and fatty acid uptake [14].

In the present study, we evaluated if whether intrauterine protein restriction in combination with overfeeding during lactation would cause adult-onset obesity and the development of metabolic disorders.

## Methods and materials

### Animals and diets

The experimental procedures involving rats were performed in accordance with the guidelines of the Brazilian Society of Science in Laboratory Animals (SBCAL) and were approved by the ethics committee at the Federal University of Mato Grosso (Process nº 23108.021972/10-4). Male and virgin female Wistar rats (aged 85–90 d) were obtained from the university’s breeding colony. Mating was prompted by housing one male with four females overnight, and pregnancy was confirmed by examining vaginal smears for the presence of sperm. Pregnant females were randomly separated during pregnancy, they were fed isoenergetic diets containing 17% protein and 3.9 kcal/g (control [C]); or 6% protein and 3.8 kcal/g (low protein [LP]). Casein served as the protein source. After birth, litters of male rats from dams fed with C and LP diets were adjusted to a litter size of four (CO and LO groups, respectively) to induce early postnatal overfeeding or eight (CC and LC groups, respectively) to induce normal feeding. After weaning (week 4), the offspring of all the litter groups were fed a diet containing 12% protein (maintenance diet) and 3.74 kcal/g, until they were 90 d old. All of the diets used in the present study are described in Table 1[15]. In the low protein and maintenance diets, adjustments were made to equalize the proportion of L-cystine in relation to the casein content offered in the control diet. After weaning the animals were housed four per cage. The rats had free access to food and water, and they were kept under standard lighting conditions (12 h light–12 h dark cycle) at 24°C throughout the experimental period.

**Table 1:** Composition of the control growth, low-protein and control maintenance diets

**Ingredientes (g/kg)**	**Control growth (17% protein)**	**Low-protein (6% de protein)**	**Control maintenance (12% de protein)**
Maize starch	397.0	480.0	439.42
Dextrinised maize starch	130.5	159.0	146.47
Casein (84% de protein)	202.0	71.5	132.0
Soyabean oil	70.0	70.0	70.0
Fibre	50.0	50.0	50.0
Sucrose	100.0	121.0	111.6
AIN-93G mineral mix*	35.0	35.0	35.0
AIN-93G vitamin mix*	10.0	10.0	10.0
L-Cystine	3.0	1.0	2.2
Choline bitartrate	2.5	2.5	2.5

AIN, American Institute of Nutrition.

*See [15].

### Experimental procedures

The rats were weighed at birth, after weaning, and then once a week throughout the experimental period. Recorded weekly, the weight and the naso-anal length (NAL) of the rats were used to calculate the Lee index [16]: Lee index= ^3^√weight body(g)/NAL (cm) × 1000 The food intake was recorded three times per week, and the data were expressed using absolute and relative values. To assess the relative food intake, the total food intake during the experimental period after weaning was normalized per 100g of body weight at age 90 d. Because it was not possible to evaluate all variables in the same animal, the number of individual experiments varied among the groups, but it was representative of at least three different litters.

At 90 d of age, the animals were euthanized with CO_2_ after fasting 12 h. Blood samples were collected, and serum was obtained by centrifugation to determine the lipid profile (triglycerides, total cholesterol, LDL cholesterol and HDL), blood glucose and insulin. Serum aliquots (stored at -80°C) of animals in the fed state were used to determine the serum concentrations of albumin, total protein, free fatty acid (FFA) and leptin. After a medium laparotomy, the retroperitoneal white adipose tissue (RWAT) and epididymal white adipose tissue (EWAT) were removed, and the mass of the tissues was measured. The tissues were frozen in liquid N_2_ and stored at −80°C for a subsequent analysis of their lipid content. The interscapular brown adipose tissue (IBAT) was removed, carefully cleaned to remove adhering fat and muscle, weighed and stored similarly to the white adipose tissues for a subsequent analysis of its lipid content.

### Oxygen consumption/carbon dioxide production and respiratory exchange coefficient determination

Oxygen consumption, carbon dioxide production and the respiratory exchange coefficient (RQ) were measured in fed animals by a computer-controlled, open circuit calorimeter system, the LE405 Gas Analyzer (Panlab – Harvard Apparatus, Holliston, MA, USA). Individual rats were placed in the metabolic chamber and allowed to acclimate for 4 hs prior to data collection. After acclimation the rats were singly housed in clear respiratory chambers and room air was circulated through the chambers at a flow rate of 0.8 l/min. The air flow within each chamber was monitored by an Air Supply and Switching sensor (Panlab – Harvard Apparatus). The gas sensors were calibrated before beginning the experiments with primary gas standards containing known concentrations of O_2_ and CO_2_ (Air Liquid, São Paulo, Brazil). The analyses were performed for a 12 h dark period in each chamber. Two hours were discounted to accommodate the dark-to-light or light-to-dark transition periods. Therefore, each rat was evaluated for 10 h. The outdoor air reference values were sampled after every four measurements. Sample air was sequentially passed through O_2_ and CO_2_ sensors to determine the O_2_ and CO_2_ content; from these data, measures of oxygen consumption (VO_2_) and carbon dioxide production (VCO_2_) were estimated. The VO_2_ and VCO_2_ were calculated with Metabolism 2.2v software and expressed in mL.h-1.g-1, based on the Withers equation. The RQ was calculated as VCO_2_/VO_2_. Energy expenditure (EE) (kilocalories per kilogram per h; heat production) was calculated using a rearrangement of the Weir equation ((3.851+1.232 × RQ) × VO2) [17,18].

### Evaluation of spontaneous locomotor activity

Spontaneous locomotor activity was evaluated during a 12 h dark period. Information on motility was gathered using two horizontal axes and, a computer-controlled linear detection system from Harvard Instruments (Panlab, Holliston, MA, USA).

### Hormones and biochemical parameters

The serum hormone levels were measured with an ELISA, using kits designed specifically for rats (GDV programmable MPT Reader DV 990BV6). Plasma or serum leptin was determined with a radioimmunoassay (RIA) kit (Linco Research, St. Louis, Missouri USA). Plasma or serum insulin was determined with an RIA kit (Millipore, Massachusetts. USA). Serum triglycerides, total cholesterol (TC), high-density lipoprotein cholesterol (HDL), low-density lipoprotein cholesterol (LDL) were analyzed by an enzymatic method using BT-3000 Plus analyzer (Wiener labs, Rosario, Argentina). The Castelli indices I and II that correlate with atherogenicity were obtained using the following formulas [19]:

(1) CastelliindexI=TC/HDL

(2) CastelliindexII=LDL/HDL

Serum glucose (in mg/dL), was determined using a monitor with test strips to measure blood glucose (glucometer) (*Accu-Chek®,* Roche Diagnostics, Germany). FFA levels were determined by a spectrophotometric method using kits by Wako Chemicals USA, Inc. The homeostasis model assessment of insulin resistance (HOMA-IR) was used as the physiological index of insulin resistance. The HOMA-IR was calculated from the fasting glucose and the fasting insulin concentrations using the following formula:

(3) $$\mathrm{HOMA}-\mathrm{IR}\;=\left(\mathrm{fasting}\;\mathrm{insulin}\;\left(\mathrm{pmo}1/1\right)\right)\;\times \;\left(\mathrm{fasting}\;\mathrm{glucose}\;\left(\mathrm{mmo}1/1\right)\right)/22.5$$

### Intraperitoneal glucose-tolerance test

After a 12 h fast, glucose (200 g/l) was administered intraperitoneally at a dose of 2 g/kg body weight. Blood samples were obtained from the cut tip of the tail 0, 30, 60 and 120 min later to determine serum glucose and insulin concentrations. The glucose and insulin responses during the intraperitoneal glucose tolerance tests were calculated by estimating the total area under the glucose curves, using the trapezoidal method [20].

### Intraperitoneal Insulin-tolerance test

A subcutaneous insulin tolerance test was performed on the 83 day-old male rats, after a 12 h fast. The insulin tolerance test consisted of a bolus injection of insulin (300 mU/kg body weight) beneath the dorsal skin of the fasting rat. Blood samples were obtained from the cut tip of the tail 0, 5, 10 and 15 min later to measure glucose levels. The rate constant for the elimination of serum glucose was calculated using the formula 0.693/*t*1=2, where *t*1=2 is half-life. The serum glucose half-life was calculated from the slope of a least-square analysis of the serum glucose concentrations from 0–15 min after the subcutaneous injection of insulin, when the serum glucose concentrations declined linearly [21].

### Lipid content

The lipid contents of the IBAT, RWAT and EWAT were determined by the gravimetric method following chloroform–methanol (2:1) extraction, described by Folch et al. [22]).

### Statistical analysis

The results are expressed as the mean values with standard errors for the number of rats indicated. Unpaired t tests were used to analyze the size of the litters and the body weight of the newborn rats. Bartlett’s test for the homogeneity of variances was initially used to determine whether the data complied with the assumptions necessary for a parametric ANOVA. When necessary, the data were log-transformed to correct for the variance in heterogeneity or non-normality [23]. A two-way ANOVA (the effect of previous nutritional status and overfeeding) was used to compare the data from the CC, CO, LC and LO groups. When necessary, these analyses were complemented by the least significant difference test to determine the significance of the individual differences. P<0·05 indicated statistical significance. All statistical analyses were conducted using the Statistic software package (Stat-soft).

## Results

### Measurements of animal weight, increase in body weight, Lee’s index and food intake

The litter size in the C and LP groups of pups was similar, but the body weight of the newborn LP rats was lower than that of the C rats (data not shown). The increase in body weight from birth to weaning (0–28 d) was similar in the LO and CO groups; in both groups, body weight was higher than in the LC and CC groups. The lowest increase in body weight was observed in the LC group (*P*<0.01). At the time of weaning, the LO group had a similar body weight to the CO and CC groups, which had higher body weights than the LC rats. However, the body weight of the CO group was higher in relation to the CC group (*P*<0.01). At the time of weaning, the Lee index was lower in the CO and LO groups than in the CC and LC groups (F_1,20_=63.48, *P*<0.01). However, the Lee indices for the LC and LO groups were higher when compared to the CC and CO groups (F_1,20_=243.00, *P*<0.001). The increases in body weight from weaning to the end of the experiment (28–90 d) and from birth to the end of the experiment (0–90 d) were significantly higher in the CO and LO groups than in the CC and LC groups (F_1,27_=11.91, *P*<0.001 and F_1,27_=29.92, *P*<0.0001, respectively). Similarly, the nutritional status was higher in the CO and LO groups, compared to the CC and LC groups (F_1,20_=243.00; *P*<0.001). At the end of the experimental period, the mean body weight and the mean Lee index of the rats from the CO and LO groups were higher than the corresponding values for the rats from the CC and LC groups (F_1,20_=22.08, *P*<0.001 and F_1,20_=7.22, *P*<0.02, respectively). The absolute food intake and relative food intake were higher in the CO and LO groups, compared to the CC and LC groups (F_1,27_=8.59, *P*<0.01 and F_1,27_=5.97, *P*<0.02, respectively). The relative food intake in the LC and LO groups was higher than in the CC and CO groups (F_1,27_=24.11, P<0.0001) (Table 2).

**Table 2:** Somatic profile of rats maintained with the control (CC and LC) or overfeeding diets (CO and LO)

**Variables**	**Groups**											
	**CC**			**CO**			**LC**			**LO**		
	**n**	**Mean**	**SEM**	**n**	**Mean**	**SEM**	**n**	**Mean**	**SEM**	**n**	**Mean**	**SEM**
Increment in body weight 0-28d (g)	6	56.9^b^	1.1	6	69.7^a^	2.4	6	41.2^c^	1.8	6	65.7^a^	1.0
BW at weaning (g)	6	61.3^b^	1.5	6	74.4^a^	2.8	6	45.7^c^	12.4	6	68.9^ab^	1.8
Lee index at weaning	6	108.5	0.8	6	102.5^#^	0.8	6	119.5^*^	0.8	6	114.0^*#^	0.6
Increment in body weight 28-90d (g)	6	317.4	4.8	6	359.4^#^	10.6	6	324.6	13.9	6	333.3 ^#^	6.1
Increment in body weight 0-90d (g)	6	374.4	4.8	6	429.1^#^	9.9	6	365.9	14.2	6	339.0^#^	6.2
Final BW (g)	6	372.3	6.1	6	422.8^#^	8.2	6	371.5	13.9	6	413.7^#^	9.5
Final Lee index	6	284.1	2.8	6	293.6^#^	3.6	6	285.4	1.2	6	289.3^#^	1.6
Absolute food intake (g)	8	1030	16.6	8	1107^#^	0.1	7	1021	61.1	8	1125^#^	0.1
Relative food intake (g/100 g BW)	8	253.6	7.1	8	272.8^#^	4.7	7	292.6^*^	12.6	8	312.47^#*^	5.6

BW body weight.

^abc^ Mean values with unlike superscript letters were significantly different (P<0·05; two-way ANOVA followed by least significant difference (LSD) test).

^*^ Mean values were significantly different from those of the control rats (P<0·05; two-way ANOVA).

^#^ Mean values were significantly different from those of the non-overfeeding rats (P<0·05; two-way ANOVA).

^n^ Numbers of rats.

### Oxygen consumption (VO_2_), carbon dioxide production (VCO_2_), respiratory quotient (RQ), energy expenditure (EE) and spontaneous activity

Carbon dioxide production, oxygen consumption and energy expenditure were higher in the rats from the CO and LO groups than in the rats from the CC and LC groups (F_1,12_=15.15, P<0.002; F_1,12_=13.79, *P*<0.001; F_1,12_=15.09, *P*<0.01, respectively) and lower in the LC and LO groups than in the CC and CO groups (F_1,12_=5.84, *P*<0.03; F_1,12_=6.10, *P*<0.02; F_1,12_=5.90, *P*<0.05, respectively). The respiratory quotient (RQ) did not differ among groups. Spontaneous activity was lower in the LC and LO groups, compared to the CC and CO groups (F_1,12_=9.86, *P*<0.01) (Table 3).

**Table 3:** **Oxygen consumption (O**_**2**_**), carbon dioxide production (CO**_**2**_**), respiratory quotient (RQ) and spontaneous activity was measured of rats maintained with the control (CC and LC) or overfeeding diets (CO and LO)**

**Variables**	**Groups**											
	**CC**			**CO**			**LC**			**LO**		
	**n**	**Mean**	**SEM**	**n**	**Mean**	**SEM**	**n**	**Mean**	**SEM**	**n**	**Mean**	**SEM**
CO_2_ production (ml/mim/kg 0,75)	6	56.9	1.3	6	63.2^#^	1.3	6	52.5^*^	1.5	6	59.4^*#^	1.5
O_2_ consumption (ml/mim/kg 0,75)	6	67.9	1.5	6	75.6^#^	1.8	6	62.9^*^	1.2	6	70.5^*#^	2.0
RQ (VCO_2_/VO_2_)	6	0.84	0.1	6	0.84	0.1	6	0.83	0.1	6	0.84	0.1
Activity	6	6609	368	6	7223	515	6	5534^*^	563	6	4683^*^	407

^*^ Mean values were significantly different from those of the control rats (P<0·05; two-way ANOVA).

^#^ Mean values were significantly different from those of the non-overfeeding rats (P<0·05; two-way ANOVA).

^n^ Numbers of rats.

### Measurements of weight and lipid content of brown adipose tissue and retroperitoneal white adipose tissues

The weight and lipid content (g/total tissue) of the IBAT were significantly lower in the CO and LO groups than in the CC and LC groups (F_1,20_=8.57, *P*<0.001 and F_1,20_=10.58, *P*<0.001, respectively) and higher for the rats from the LC and LO groups, compared with the rats from the CC and CO groups (F_1,20_=5.09, *P*<0.02 and F_1,20_=19.67, *P*<0.001, respectively). However, the lipid content of the IBAT (g/100 g tissue) was higher in the LC and LO rats than in the CC and CO rats (F_1,20_=5.04, *P*<0.02). The absolute RWAT and EWAT weights and as well as the lipid content (g/total tissue) in these deposits, were higher in the CO and LO rats than in the CC and LC rats (F_1,20_=14.71, *P*<0.001; F_1,20_=6.38; *P*<0.05 and F_1,26_=5.89, *P*<0.02; F_1,27_=6.09; *P*<0.02, respectively). The relative weight of EWAT (mg/g body weight) was similar in the LO, LC and CO groups and those weights were higher in relation to the CC group (P<0.02). The relative weight of RWAT (mg/g body weight) was significantly higher in the CO and LO groups than in the CC and LC groups (F_1,20_=4.60, *P*<0.05) as well as in rats from the LC and LO groups compared with the rats from the CC and CO groups (F_1,20_=9.25, *P*<0.01). The lipid content in the RWAT (g/100 g tissue) and EWAT (g/100 g tissue) did not differ among the groups (Table 4).

**Table 4:** Weight and lipid content in the white and brown adipose tissues (BAT) of rats maintained with the control (CC and LC) or overfeeding diets (CO and LO)

**Variables**	**Groups**											
	**CC**			**CO**			**LC**			**LO**		
	**n**	**Mean**	**SEM**	**n**	**Mean**	**SEM**	**n**	**Mean**	**SEM**	**n**	**Mean**	**SEM**
Weight IBAT (g)	6	0.468	0.1	6	0.391^#^	0.1	6	0.652^*^	0.1	6	0.501^*#^	0.1
Lipid content IBAT (g/total tissue)	6	0.20	0.1	6	0.16^#^	0.1	6	0.34^*^	0.1	6	0.23^*#^	0.1
Lipid content IBAT (g/100g tissue)	6	48.7	3.2	6	50.1	3.4	6	61.3^*^	2.1	6	52.5^*^	4.4
Weight RWAT (g)	6	9.1	0.5	6	12.1^#^	0.8	6	10.1	0.4	6	12.5^#^	0.9
Weight RWAT (g/ 100g BW)	6	2.5	0.1	6	2.6^#^	0.3	6	2.7^*^	0.1	6	3.10^*#^	0.1
Lipid content RWAT (g/total tissue)	8	3.5	0.3	7	4.9^#^	0.4	7	3.7	0.4	8	4.1^#^	0.3
Lipid content RWAT (g/100g tissue)	8	39.6	2.1	7	38.0	1.5	7	38.6	2.5	8	33.6	1.3
Weight EWAT (g)	6	6.6	0.5	6	8.7^#^	0.7	6	8.3	0.7	6	9.7^#^	0.8
Weight EWAT (g/ 100g BW)	6	1.8^b^	0.1	6	2.2^a^	0.1	6	2.4^a^	0.1	6	2.2^a^	0.2
Lipid content EWAT (g/total tissue)	8	2.8	0.1	8	3.3^#^	0.2	7	2.7	0.3	8	3.2^#^	0.2
Lipid content EWAT (g/100g tissue)	8	35.7	0.8	8	36.4	0.6	6	34.3	1.2	7	34.3	0.7

BW, body weight; RWAT, retroperitoneal white adipose tissue; EWAT, epididymal white adipose tissue.

^ab^ Mean values with unlike superscript letters were significantly different (P<0·05; two-way ANOVA followed by least significant difference (LSD) test).

^*^ Mean values were significantly different from those of the control rats (P<0·05; two-way ANOVA).

^#^ Mean values were significantly different from those of the non-overfeeding rats (P<0·05; two-way ANOVA).

^n^ Numbers of rats.

### Biochemical parameters

The triglycerides levels were lower in the rats from the CO and LO groups, compared with the rats from the CC and LC groups (F_1,19_=16.64; *P*<0.001), but triglycerides were higher in the LC and LO rats than in the CC and CO rats (F_1,19_=6.43; *P*<0.02). The LDL-cholesterol, HDL-cholesterol, total cholesterol and Castelli I did not differ among the groups. However, the Castelli II values were higher in the CO group than in the LO, CC and LC groups (*P* <0.05). The serum FFA concentrations were lower in the CO and LO rats than in the CC and LC rats (F_1,20_=4.89; *P*<0.05) (Table 5). The serum total protein and albumin concentrations did not differ among groups (data not shown).

**Table 5:** Biochemical parameters of rats maintained with the control (CC and LC) or overfeeding diets (CO and LO)

**Variables**				**Groups**								
	**CC**			**CO**			**LC**			**LO**		
	**n**	**Mean**	**SEM**	**n**	**Mean**	**SEM**	**n**	**Mean**	**SEM**	**n**	**Mean**	**SEM**
Triglycerides (mg/dl)	6	181.4	16.4	5	84.2^#^	7.8	6	190.9^*^	12.2	6	156.5^* #^	21.9
LDL- cholesterol (mg/dl)	6	26.6	4.2	6	33.0	3.4	6	30.8	4.7	6	30.5	2.3
HDL- cholesterol (mg/dl)	6	23.6	0.6	6	20.1	1.9	6	24.6	1.2	6	23.5	0.9
Total cholesterol (mg/dl)	6	86.5	3.3	6	86.3	12.5	6	93.6	7.4	6	85.2	4.4
Castelli I	6	3.7	0.1	6	3.9	0.1	6	3.8	0.2	6	3.6	0.1
Castelli II	6	1.1^b^	0.2	6	2.1^a^	0.2	6	1.2 ^b^	0.2	6	1.3^b^	0.1
FFA (μmol/l)	6	0.99	0.1	6	0.72^#^	0.1	6	0.88	0.0	6	0.84^#^	0.0

FFA, free fatty acid.

^abc^ Mean values with unlike superscript letters were significantly different (P<0·05; two-way ANOVA followed by least significant difference (LSD) test).

^*^ Mean values were significantly different from those of the control rats (P<0·05; two-way ANOVA).

^#^ Mean values were significantly different from those of the non-overfeeding rats (P<0·05; two-way ANOVA).

^n^ Numbers of rats.

### Metabolic and hormonal parameters

Basal serum glucose levels were similar among the groups. However, serum insulin levels and HOMA-IR were higher in the CO and LC groups than in the CC and LO groups. Additionally, in the LO group, these parameters were lower than in the CC group (*P*<0.05). No differences were observed among the leptin concentration levels of the groups. The total area under the glucose curve for the CO and LO rats was higher than for the CC and LC rats (F_1,21_=13.25, *P*<0.01). The total area under the insulin curve was similar in the CO and LC groups, and both were higher than the corresponding values for the LO and CC groups (*P*<0.01) (Table 6).

**Table 6:** **Fasting serum glucose and insulin concentration, total area under the glucose (AUG) and insulin (AUI) curves, glucose disappearance ratio (K**_**itt**_**) obtained from the intraperitoneal insulin tolerance test and HOMA-IR of rats maintained with the control (CC and LC) or overfeeding diets (CO and LO)**

**Variables**				**Groups**								
	**CC**			**CO**			**LC**			**LO**		
	**n**	**Mean**	**SEM**	**n**	**Mean**	**SEM**	**n**	**Mean**	**SEM**	**n**	**Mean**	**SEM**
Basal glucose (mmol/l)	7	7.0	0.1	7	7.1	0.1	3	6.8	0.0	6	7.0	0.1
Basal insulin (pmol/l)	7	14.8^b^	0.8	7	35.3^a^	2.3	3	34.6 ^a^	1.3	6	3.3 ^c^	0.7
AUG (mmol/L. 120 min)	7	2065	31	4	2278^#^	95	7	2075	27	7	2183^#^	52
AUI (pmol/L. 120 min)	7	56 ^b^	4	4	200 ^a^	53	7	173 ^a^	44	7	78 ^b^	3
K_itt_ (%/min)	8	0.28 ^b^	0.2	8	2.79 ^a^	0.6	7	3.05 ^a^	0.3	8	3.13 ^a^	0.4
HOMA-IR	7	4.6 ^b^	0.3	7	11.1 ^a^	0.7	3	10.4 ^a^	0.4	6	1.0 ^c^	0.2
Leptin (μU/ml)	5	12.1	1.6	5	11.8	1.2	5	10.8	0.4	5	10.2	0.5

^abc^ Mean values with unlike superscript letters were significantly different (P<0·05; two-way ANOVA followed by least significant difference (LSD) test).

^*#^ Mean values were significantly different from those of the control rats (P<0·05; two-way ANOVA).

^n^ Numbers of rats.

## Discussion

In the present study, restricting protein in the diets of rats during fetal life led to a low birth weight. Overfeeding during lactation, induced by small litter size resulted in a higher body weight at weaning and in adulthood for the offspring, regardless of the protein content of the mother’s diets during pregnancy. However, based on Lee’s index, the protein restriction during intrauterine life resulted in obesity at weaning, independent of postnatal nutrition. It is well established that early overfeeding induces a dramatic increase in body weight during the suckling period [7,24,25].

Postnatal overfeeding and normal nutrition after weaning corrected the features typical of protein malnutrition (hypoalbuminemia and low serum total protein), and resulted in a higher final body weight, Lee’s index, and visceral fat mass. An increased fat mass and adipocyte number has been observed in a similar animal model [26-28]. These animals were shown to exhibit enhanced 11-β-hydroxysteroid dehydrogenase type 1 (11β- HSD1) mRNA in adipose cells, an enzyme that converts cortisone to its active form of cortisol [25]. It has been suggested that exacerbation of adipose tissue in obese rodents and humans occur due to elevated glucocorticoid production as a result of increased 11β- HSD1 [29,30]. We verified that prenatal protein restriction did not alter body weight per se but increased visceral fat deposits. However, prenatal protein restriction combined with postnatal overfeeding did not modify the body weight, Lee’s index, and visceral pad weight and content. Therefore, our results are in disagreement with human studies that showed that growth-restriction *in utero* followed by postnatal catch-up growth contributes to increased central fat mass [31].

Obesity is the result of an imbalance between energy intake and energy expenditure; excess energy is stored as fat whenever energy intake exceeds energy expenditure. Hyperphagia, among other factors, seems to have contributed to the excessive body weight gain that occurred in our overfed groups. Interestingly, an increased appetite was also observed in the prenatal protein restricted rats, when the food consumption data were normalized for body weight. This finding contradicts the observation that in rodents, unlike in humans, the development of the hypothalamic appetite regulatory system occurs predominantly after birth [32]. Early postnatal overnutrition, induced by small litter size [6,7,24,25,27,33], causes persistent hyperphagia and alters the hypothalamic energy homeostasis mechanism in adulthood [2]. The hypothalamus is the primary center in the brain that regulates food intake and body weight homeostasis [34]. It is subject to the influence of several peripheral factors, including circulating levels of leptin [35], that which are proportional to the total fat mass [36]. We verified that the circulating leptin levels were not modified by early protein restriction or by overfeeding during lactation. Moreover, the serum leptin concentrations were not equivalent to the absolute and relative weight and lipid content of the RWAT and EWAT. These results were not surprising, at least for postnatal overfed rats, because, hyperleptinemia occurs only at weaning, when subcutaneous adipose tissue is increased [7] and leptin is produced mainly by subcutaneous adipose tissue [37,38]. It is noteworthy that the hyperphagia in rats exhibiting unaltered serum leptin levels, as observed in the animals that were submitted to intrauterine protein restriction or early postnatal overfeeding, suggests of leptin resistance.

Spontaneous activity levels, O_2_ consumption and CO_2_ production were reduced in the animals submitted to prenatal protein restriction that exhibited a lean profile. In contrast, in the overfed rats that showed a phenotype of obesity, the activity levels did not change, but the O_2_ consumption and CO_2_ production increased. The higher O_2_ consumption, CO_2_ production and energy expenditure in the overfed animals could be explained by higher body weight. One possible explanation for the different phenotypes exhibited by our equally hyperphagic animals is the different levels of facultative thermogenesis. This hypothesis is reinforced by the diverse profile of IBAT weight and lipid content in the overfed and protein restricted rats. It has been demonstrated that IBAT thermogenic activity is accompanied by a simultaneous elevation in fatty acid synthesis and IBAT weight [39,40]. Thus, we suggest that IBAT thermogenic activity was enhanced by the prenatal protein restriction and suppressed (decreased) by overfeeding during lactation. Reduced responsiveness to sympathetic stimulation and down-regulated adrenoreceptors in WAT, commonly observed in obese rodents [41-43], could have contributed to the increase in lipid deposits in our overfed animals. This effect is manifested by a decrease in fatty acid mobilization [44], and our overfed animals exhibited reduced FFA levels. FFA release from adipose tissue is also down-regulated by fasting hyperinsulinemia [45] and could, therefore, be another factor contributing to low FFA levels and reduced fatty acid mobilization, at least in the CO group.

Independent of prenatal nutrition, postnatal overfeeding resulted in impaired glucose tolerance. Curiously, the overfed groups (CO and LO) exhibited opposite basal and stimulated serum insulin profiles and, varying hepatic insulin sensitivity, but similar peripheral insulin sensitivity. These observations are based on the HOMA-IR and K_itt_ values, respectively. Insulin sensitivity estimated by HOMA-IR predominantly expresses the ability of basal insulin to suppress hepatic glucose production in a fasting state, whereas K_itt_ is more influenced by the disposal of glucose after insulin application, reflecting peripheral insulin resistance [46]. Increase in the body weight and Lee’s index were not accompanied by peripheral insulin resistance, and the increase in visceral fat depots was not a determinant of liver insulin resistance. It appears that hepatic insulin resistance was determined by the serum insulin concentration, corroborating the observation that insulin sensitivity and insulin secretion are reciprocal and inversely related [47]. Another adequate explanation for the unrelated visceral fat deposits and liver insulin resistance is the mild increase in visceral adiposity observed in our overfed animals. The LC group had relative RWAT and EWAT weights and lipid contents similar to the LO group, however only the LC group exhibited hepatic insulin resistance. Remarkably, whereas prenatal protein restriction and overfeeding during lactation resulted in hyperinsulinemia in the basal state and after glucose challenge, fetal protein restriction combined with overfeeding during lactation, produced a state of basal insulin deficiency. Although the insulin deficiency was counteracted by the increase in peripheral and hepatic insulin sensitivity in the LO group, it was not enough to warrant glucose homeostasis. The insulin AUC after glucose challenge was similar in the LO and CC groups. However, these results are not indicative of normal secretion, because the insulin AUC after GTT provides an idea of the amount of insulin that acts on the tissues but cannot provide information on the dynamic of the hormone in terms of secretion, extraction and clearance [48]. In addition to the alterations in glucose metabolism, this study found an increased cardiovascular risk in CO rats, because the Castelli II index was elevated in that group. Prenatal protein restriction also conferred an increased cardiovascular risk, considering the high triglyceride levels.

In conclusion, our results suggest that overfeeding during lactation increased visceral fat and body mass, altered lipid metabolism and contributed to glucose intolerance by distinct mechanisms. When postnatal overfeeding was combined with normal intrauterine nutrition, impaired glucose tolerance resulted from insulin resistance. When postnatal overfeeding was combined with intrauterine protein restriction, it resulted in deficient pancreatic function.

## Abbreviations

AUC: Area under the curve; AIN: American Institute of Nutrition; BW: Body weight; CC: Offspring born from mothers fed a control diet whose litter was adjusted by eight pups during lactation; CO: Offspring born from mothers fed a control diet whose litter was adjusted by four pups during lactation; EE: Energy expenditure; EWAT: Epididymal white adipose tissue; FFA: Free fatty acid; HDL: High-density lipoprotein; IGTT: Intraperitoneal glucose tolerance test; IBAT: Interscapular brown tissue; Kitt: Glucose disappearance ratio; LC: Offspring born from mothers fed a low protein diet whose litter was adjusted by eight pups during lactation; LDL: Low-density lipoprotein; LO: Offspring born from mothers fed a low protein diet whose litter was adjusted by four pups during lactation; NAL: Naso-anal length; RIA: Radioimmunoassay; RQ: Respiratory quotient; RWAT: Retroperitoneal white adipose tissue; TC: Total cholesterol; VCO2: Carbon dioxide production; VO2: Oxygen consumption; Δ**G**: Total area under the glucose curve; Δ I: Total area under the insulin curve; 11β- HSD1: 11β-Hidroxysteroid dehydrogenase type 1.

## Competing interests

The authors declare that they have no competing interests.

## Authors’ contributions

GVPC Participated in the all of the study included the drafted of the manuscript. FRC, JZF, MST, LMIS and AAP participated in the experimental phase. SRLR participated in the experimental phase and the statistical analysis. MQL performed the statistical analysis. MHGGS participated in the preparation of manuscript. MSFM participated in the design, coordination of the study, and drafted the manuscript. All authors read and approved the final manuscript. 
